# Meteorological Approach in the Identification of Local and Remote Potential Sources of Radon: An Example in Northern Iberian Peninsula

**DOI:** 10.3390/ijerph20020917

**Published:** 2023-01-04

**Authors:** Miguel Ángel Hernández-Ceballos, Natalia Alegría, Igor Peñalva, Jose Miguel Muñoz, Alejandro De la Torre, Fernando Legarda, Giorgia Cinelli

**Affiliations:** 1 Department of Physics, University of Cordoba, 14071 Córdoba, Spain; 2 Department of Energy Engineering, University of the Basque Country, 48013 Bilbao, Spain; 3 Department of Industry, Basque Government, 01003 Vitoria, Spain; 4 Laboratory of Observations and Measurements for the Climate and the Environment, National Agency for New Technologies, Energy, and Sustainable Economic Development (ENEA), 21027 Ispra, Italy

**Keywords:** ^222^Rn, daily cycle, air masses, TSA and PSCF, Bilbao

## Abstract

This paper presents a meteorological approach to identify local and remote sources driving the variability of surface daily radon concentrations. To this purpose, hourly ^222^Rn concentration and surface meteorological measurements, and air mass trajectories at Bilbao station (northern Iberian Peninsula) during the period 2017–2018 have been taken as reference. To investigate the potential transport pathways and potential ^222^Rn sources, the backward trajectory cluster analysis, trajectory sector analysis (TSA), and potential source contribution function (PSCF) are applied. On average, the diurnal ^222^Rn cycle shows the expected behaviour, with larger concentrations during the night and minimum concentrations during the daylight hours, with differences in the seasonal amplitudes. According to daily differences between maximum and baseline values, ^222^Rn daily cycles were grouped into six groups to identify meteorological conditions associated with each amplitude, and potential source areas and transport routes of ^222^Rn over Bilbao. The trajectory cluster and the TSA method show that the main airflow pathways are from the south, with small displacement, and the northeast, while the analysis of surface wind speed and direction indicates that the highest amplitudes of ^222^Rn concentrations are registered under the development of sea-land breezes. The PSCF method identified south-western and north-eastern areas highly contributing to the ^222^Rn concentration. These areas are confirmed by comparing with the radon flux map and the European map of uranium concentration in soil. The results have demonstrated the need in combining the analysis of local and regional/synoptic factors in explaining the origin and variability of ^222^Rn concentrations.

## 1. Introduction

The radioactive noble gas radon (hereby, ^222^Rn), a colourless, odourless, and chemically inert gas is part of the natural decay chain of ^238^U and originates directly from the decay of ^226^Ra, practically ubiquitous in rocks and soils. ^222^Rn is released from soils and rocks and reaches the atmosphere through three main processes: (i) Emanation -release from the solid mineral grains to the filled pores; (ii) Transport of the emanated radon through the pores of the soil/rock into the ground surface; (iii) Exhalation the radon released to the atmosphere and the exhalation rate is defined as the amount of radon released per surface and time unit [1].

Due to the relatively long half-time (T_1/2_ ≈ 3.8 d), it tends to accumulate in buildings reaching elevated concentrations in indoor air (up to thousands Bq/m^3^), which, represents a health hazard for the inhabitants. Indeed, the inhalation of ^222^Rn and its progenies is the largest source of public exposure to naturally occurring radioactivity [2]. It is responsible for half of the natural dose received by the global population [2] and it is a major cause of lung cancer after smoking [3]. On the other hand, radiation exposure from inhalation of outdoor ^222^Rn and its progeny is less compared to those indoors and it is known to have no major impact on health [3]. ^222^Rn released in the atmosphere is quickly diluted reaching a concentration in the range between 1 Bq/m^3^ and 100 Bq/m^3^ [4]. It depends not only on the magnitude of the release rate from the soil but also on atmospheric mix phenomena. However, outdoor concentrations can reach hazardous levels in regions with high natural radiation or NORM—Naturally Occurring Radioactive Material sites (i.e., phosphogypsum piles [5]).

Although outdoor ^222^Rn does not represent a significant health risk to the general population, it plays an important role in scientific research. For this reason, it is routinely measured in many parts of the world due to its use to analyse different research topics, such as (1) to study atmospheric transport and mixing processes within the planetary boundary layer [6], (2) to characterise air mass history and fetch at remote sites [7], (3) to indirectly estimate greenhouse gas (GHG) fluxes using the Radon Tracer Method (RTM) [8,9] and in radiation protection the estimation of radon priority areas (RPA) [10], (4) to analyse residues from certain NORM industries [11], and (5) to characterize radon wash-out peaks in the ambient dose rate data, which are exchanged in the EURDEP early warning system for radiological/nuclear accidents (https://remon.jrc.ec.europa.eu/About/Rad-Data-Exchange, accessed on 14 October 2022). The EMPIR project 19ENV01 TraceRadon (http://traceradon-empir.eu/, accessed on 1 October 2022), which started in 2020, represents one example of research focused on atmospheric radon and radon flux. The project aims to improve traceable low-level radon activity concentration and radon flux measurements and to provide the necessary infrastructure for measuring both [12].

Considering the relevance of ^222^Rn measurements in different scientific disciplines, the analysis and understanding of the day-to-day variability and the identification of potential sources are needed. In this sense, the characterization of the variability of outdoor ^222^Rn concentrations in the boundary layer results from a combination of local and regional/synoptic meteorological factors [7,13]. An increase in atmospheric pressure, rainfall, and snowfall decrease the radon flux whereas an increase in wind speed or temperatures increases it, while, on the contrary, the contribution from remote sources is strongly influenced by the transport pattern affecting the regions [14]. The influence of each contribution can be evaluated by analysing both meteorological observation data and model results [11,15].

In this context, this study aims to investigate the ^222^Rn activity concentrations, characterise the transport pathways, and identify potential sources of ^222^Rn in Bilbao over a continuous time span ranging from 2017 to 2018 by means of meteorological and ^222^Rn measurements, and meteorological methods. The local footprint has been analysed using the wind and precipitation data, while the regional footprint analysis, which offers information about the synoptic atmospheric circulation, has been performed by using the HYSPLIT model by calculating backward trajectories. In this sense, air mass back trajectory analysis is frequently used to solve inverse pollutant transport by estimating the path of an air parcel backward in time at a receptor site by means of different methodologies [16,17]. In the present study, the trajectory cluster analysis and trajectory sector analysis (TSA) are used to identify transport pathways, as well as the potential source contribution function (PSCF), which is used to investigate potential sources [18,19]. To finalize, those areas identified by applying these meteorological methods are then compared with the Radon Flux Maps [20] and with the European Map of Uranium in soil available on REMONwebportal (https://remon.jrc.ec.europa.eu/About/Atlas-of-Natural-Radiation/Digital-Atlas/Uranium-in-soil/Uranium-concentration-in-soil-, accessed on 20 October 2022)

This work is organized as follows. In Section 2, we first describe the ^222^Rn and meteorological datasets, the scheme to calculate air mass trajectories, and the methodologies applied. Then, in Section 3, we present: (1) the temporal variability of the ^222^Rn; (2) the set of different daily cycles of ^222^Rn identified by comparing maximum and baselines concentrations; (3) the set of local and synoptic meteorological conditions associated with each group of daily cycles, and the location of the potential sources, and (4) the verification of these sources with those identified in the ^222^Rn exhalation rate map and the European Map of Uranium. We draw the main conclusions in Section 4.

## 2. Materials and Methods

### 2.1. Study Area

**Figure 1:**
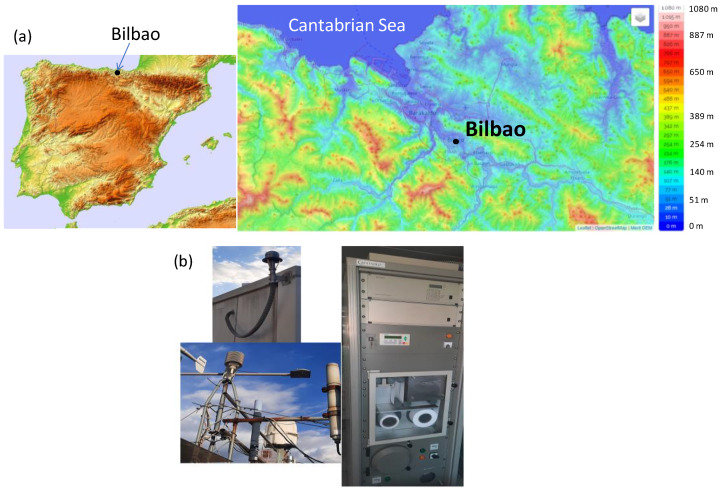
(**a**) Location of the study site in the Iberian Peninsula and the topographic map of the nearby area, and (**b**) the meteorological station and the radon measurement system at Bilbao.

The study area is Bilbao city, in the Basque province (northern Spain, Figure 1). Bilbao has located about 16 km from the Cantabric sea, following the valley of the Nervion river, which acts as a preferential channel for surface winds reaching Bilbao. The relief surrounding Bilbao is dominated by NW-SE oriented folds, with an altitude between 80 m and 700 m (Figure 1). Bilbao has a humid oceanic climate with a predominance of westerly winds. During the studied period (2017–2018), temperature averages between 11 °C in winter and 21 °C in summer, while annual precipitation averages over 830 mm, with a yearly average of 142 days with at least 1 mm. Previous analysis [21] has demonstrated that this area is typically affected by air masses from the northwest, west, north, northeast, and south, which are combined in spring and summer with the development of mesoscale circulations in surface levels, i.e., sea-land breezes.

This area could be classified as low natural radioactivity background based on the value of U contents in soil, gamma dose rate, and radon fluxes. According to the European Map of Uranium in soil (https://remon.jrc.ec.europa.eu/About/Atlas-of-Natural-Radiation/Digital-Atlas/Uranium-in-soil/Uranium-concentration-in-soil-, accessed on 10 October 2022), Bilbao area shows values of uranium between 1.6 and 2.4 mg/kg. The map of natural gamma radiation in Spain—MARNA shows values below 10 μR/h (https://www.csn.es/en/mapa-de-radiacion-gamma-natural-marna-mapa, accessed on 10 October 2022). Looking to the map obtained by [14], which provides detailed information on the ^222^Rn flux term in Spain, with a spatial resolution of 0.02 deg by downscaling the Spanish terrestrial gamma map (MARNA), this area presents an average ^222^Rn flux of less than 40–50 Bq/(m^2^ h), while in nearby areas and to the east there is a higher average of ^222^Rn flux, at around 80–90 Bq/(m^2^ h). Therefore, these low natural radioactivity levels make this region could be considered as a background station, and then, useful to identify the regional and remote sources of ^222^Rn.

### 2.2. Radon Measurements

The ^222^Rn measurements have been carried out by the Faculty of Engineering of Bilbao (43.26° N, −2.9° W). The radiological station located in Bilbao is a combined Particulate and Iodine Monitoring system Type 9850-6 (Moving Filter Monitor BAI 9100 D, IOD-131 monitor BAI9103-2 and Gamma Dose Rate Detector LB6360, Berthold trademark positioned on the roof of a building at 10 m above ground level [22]. At this height, the thoron contribution can be assumed negligible, so we do not expect fluctuations in radon/thoron decay product concentration [10]. This equipment has been checked empirically in Berthold every five years to determine any effect on the calibration factor in order to keep consistent high-quality measurements. Figure 1 shows the moving Filter Monitor BAI 9100 D with air caption and gamma dose rate detector LB6360. This Monitor BAI 9100 D consists of the following blocks: (a) a dust collection unit BAI9100D and (b) an Alpha-/Beta-Detector BAI9300AB with ZnS coated Plastic Scintillator and Preamplifier unit LB2030 for simultaneous, separate Alpha and Beta activity measurement and Radon activity concentration obtained by the Alpha Beta Pseudo-coincidence Difference (ABPD) method. The ABPD method uses the specific measurement of the Bismuth-214 decay into Polonium-214 and the Bismuth-212 decay into Polonium-212 to compensate for natural activity. A good pseudo-coincidence stage should contain a second circuit to detect radon coincidences. These should be used to compensate for the pseudo-coincidence stage for random coincidences at higher count rates to avoid overcompensation i.e., suppression of potential artificial radioactivity events. The ABPD module block diagram has two stages implemented the pseudo-coincidence stage (^214^Bi/^214^Po and ^212^Bi/^212^Po decays) and the random (A/B) coincidence stage. Gamma Dose Rate Detector LB6360 is a proportional counter with a calibration factor of 0.105 $\frac{\mu Gy}{h}per\;cps$. The sampling time is 10 min, and the calibration is completed annually with ^241^Am and ^36^Cl [22]. The methodology can introduce uncertainties in radon measurements in the order of ±10%.

The estimation of radon concentration from radon progenies requires the application of an equilibrium factor, which is known to have great variability, from 0.13 to 0.91 [10]. Measurements of the equilibrium factor were not available. So, it was decided to carry out our analysis without correcting for the equilibrium factor considering the output of the instruments, being aware that referring to ^222^Rn concentration we are referring to ^222^Rn estimated through its progeny (^214^Bi and ^214^Po).

**Figure 2:**
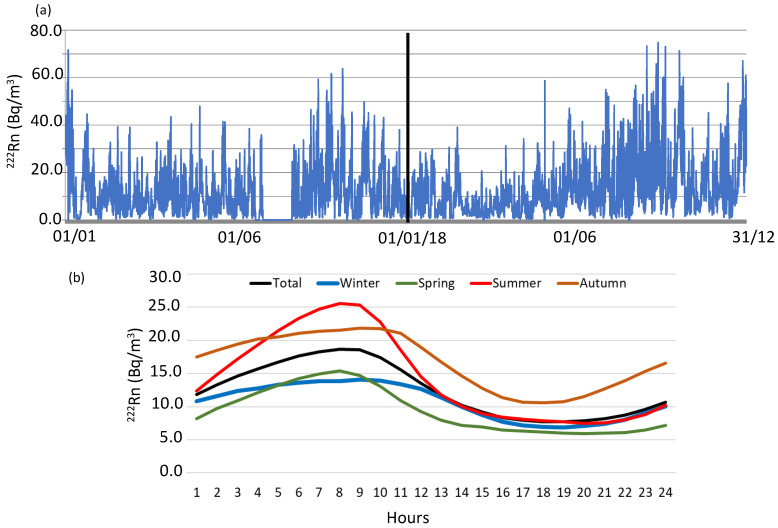
(**a**) Hourly ^222^Rn concentration evolution and (**b**) diurnal composite of average hourly radon concentrations in Bilbao during the period 2017–2018.

Working with data, it is almost impossible to acquire them without gaps due to power failure or other technical problems. Hourly measurements from 2017 to 2018 are selected based on the availability of ^222^Rn measurements (82% in 2017 and 88% in 2018), thus guaranteeing the largest statistical sample. In this case, there is one main gap in data, in August 2017 (Figure 2a).

### 2.3. Meteorological Parameters

The following meteorological variables were continuously measured in situ using a Campbell automatic weather station together with the CR1000 datalogger: temperature, relative humidity, precipitation, and wind speed and direction. These measurements were obtained at the same location as the ^222^Rn equipment, and with a time resolution of one hour. From this set of hourly measures, the quality criterion of 75% was applied to calculate daily values, i.e., at least 75% of the hourly and daily records.

### 2.4. Air Mass Trajectories and Cluster, TSA and PSFC Methods

Kinematic 3-D back trajectories were computed for a period of 2 years (2017–2018) eight times per day (at 00:00, 03:00, 06:00, 09:00; 12:00; 15:00; 18:00; 21:00 UTC). This set of backward trajectories was calculated by the HYSPLIT model, which was run with meteorological data from the Global Data Assimilation System reanalysis archive (http://ready.arl.noaa.gov/, accessed on 10 September 2022) with 1-degree global latitude-longitude projection and a temporal resolution of 6 h. These files are widely used by the scientific community to study the regional atmospheric circulation [23] and to perform a qualitative analysis of the different source regions influencing the atmospheric ^222^Rn concentrations [24,25,26]. Trajectories were computed at 100 m above ground level (agl) and with a period of 96 h. This time period agrees with the half-life of radon (3.8 days), providing unambiguous evidence of terrestrial influence over the sampling point [27,28]. This time is also used in previous studies over the Iberian Peninsula dealing with long-range transport [28,29,30,31].

Based on the set of back trajectories calculated, trajectory cluster analysis, trajectory source apportionment (TSA), and the potential source contribution function (PSCF) were applied to investigate the source-receptor relationship for the ^222^Rn concentrations in Bilbao. Details regarding the HYSPLIT k-means clustering algorithm are provided at the ARL NOAA site (ready.arl.noaa.gov/HYSPLIT.php, accessed on 1 October 2022). The TSA is a statistical approach that computes average concentrations from various directions, and hence, is useful to evaluate the effect of air masses from various directions on atmospheric concentrations of different substances [32]. In the present study, we use the calculated back trajectories and 3-h ^222^Rn concentrations to identify those regions that highly impact the receptor at Bilbao. Equations used in [33] were used to calculate the relative contribution (%C) from each of the sector 12 sectors of 30° each, numbered clockwise from 0° north.

On the other hand, the PSCF method estimates pollutant sources in upwind areas by analysing airflow trajectories and a given concentration threshold [34]. From the base that air mass alone cannot be used to identify specific source regions of pollutants [35], this method links residence time with high concentrations through a conditional probability field and provides a value to each grid cell, indicating high PSCF values the location of potential emission sources [36] in the present case, of ^222^Rn over Bilbao:

(1) $$PSCF_{ij}=\frac{n_{ij}}{N_{ij}}$$

where *N_ij_* is the total number of airflow trajectories’ endpoints that fall in the *ij^th^* grid and *n_ij_* is the total number of airflow trajectories’ endpoints for which the measured ^222^Rn concentration exceeds a given threshold in the same grid. To calculate the PSCF, the whole geographic region covered by the backward trajectories was divided into a gridded array, with the centre of Bilbao as the midpoint and containing grid cells of 0.5° × 0.5°. It is important to note that a grid with no endpoints cannot be identified as a source area in the analysis even though there are known emission sources in the grid cell, as well as studies, have demonstrated that great uncertainty exists in the calculation result when *N_ij_* is extremely small. To better reflect this uncertainty the weight function (*W_ij_*) defined in [33] was applied:

(2) $$W_{PSCF_{ij}}=W_{ij}\cdot PSCF_{ij}\;\mathrm{where}\;W_{ij}=\left\{\begin{array}{c} 1.00\;\;N_{ij}>80\;\\ 0.70\;25<N_{ij}\leq 80\\ 0.42\;15<N_{ij}\;\leq 25\\ 0.17\;N_{ij}\;\leq 15 \end{array}\right.$$

Then the value of PSCF is interpreted as the probability where the concentration of ^222^Rn higher than the creation level was related to the passage of air parcels through the *i*th cell. These cells are indicative of areas of high potential contributions for ^222^Rn in Bilbao.

## 3. Results and Discussion

### 3.1. Characterization of ^222^Rn Concentrations

Figure 2a shows the variation of hourly ^222^Rn concentration in the atmosphere of Bilbao for the period from January 2017 to December 2018. The ^222^Rn concentration with an arithmetic mean of 12.5 ± 0.2 Bq/m^3^ in 2017 and 12.4 ± 0.2 Bq/m^3^ in 2018 respectively, with a standard uncertainty of the mean equal to Sx/(N)½ being Sx the standard deviation and N the total number of measurements. Similar ^222^Rn concentrations are also registered for 90th (28.2 Bq/m^3^ and 27.2 Bq/m^3^) and 10th (2.1 Bq/m^3^ and 2.3 Bq/m^3^) percentiles. Figure 2 also shows the composite diurnal cycles for ^222^Rn concentrations during the whole sampling period. On average, the night-time maximum of the hourly concentration is 18.7 Bq/m^3^ at 8:00 UTC, which is associated with the accumulation of ^222^Rn in the lower layers of the atmosphere due to the enhancement of atmospheric stability. This process favours the accumulation of freshly exhaled radon in lower layers [36]. After this increase at night, ^222^Rn concentrations decrease during the daytime, reaching a minimum value of 7.7 Bq/m^3^ on average at 17:00–18:00 UTC. This decrease is due to solar radiation, which breaks the night-time stability and increases the vertical radon dilution.

**Figure 3:**
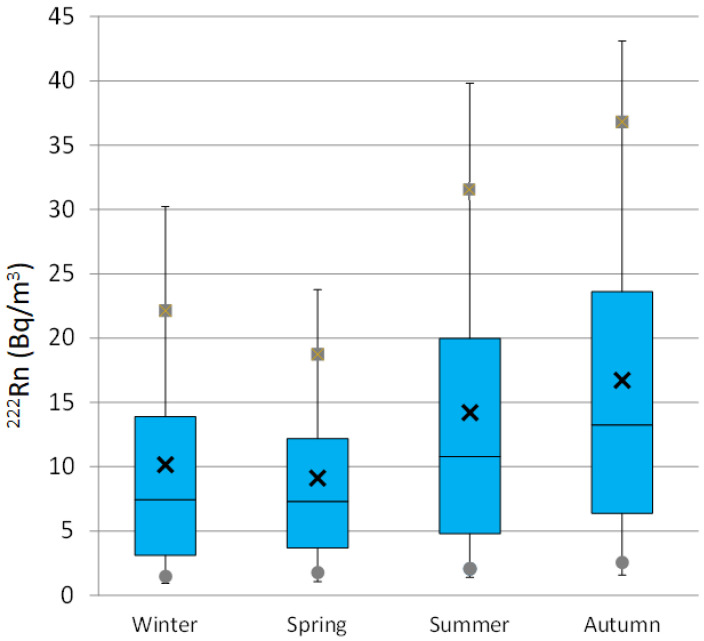
Seasonal box plots of ^222^Rn hourly concentrations at Bilbao. The rectangle represents 50% of data (interquartile range from 25th to 75th percentile), the small cross identifies the mean, the continuous horizontal line inside the rectangle identifies the median (50th percentile), the squares and circles identify the 90th and 10th percentiles respectively, and the whiskers extend between the 95th and 5th values.

This general daily cycle presents seasonal differences (Figure 2b), in line with the corresponding seasonal box-and-whisker plots displayed in Figure 3. Meteorological seasons were defined as winter, January to March, spring April to June, summer July to September, and autumn October to December. There is a large increase in the maximum daily values from Winter (14.2 Bq/m^3^) to Summer (26.2 Bq/m^3^), being similar to the minimum ones, reaching the highest in Autumn (10.2 Bq/m^3^). The period of high ^222^Rn concentrations coincides with the largest influence of stable synoptic conditions (e.g., Azores anticyclone) over the Iberian Peninsula, which favours the development of mesoscale processes, such as sea-land breezes, and hence, increasing recirculation and stagnation processes of surface air. In Figure 3, the highest hourly concentrations, close to 75 Bq/m^3^, are mainly reached from August to early October, which causes an annual pattern with low radon levels in spring months and an increase in autumn. In this sense, it is necessary to mention the considerable difference between maximum ^222^Rn concentrations and 95th percentile values in each season, ranging from 30.1 Bq/m^3^ (Winter) to 40.2 Bq/m^3^ (Summer). The maximum value tends roughly to be twofold or even threefold compared to the 95th percentile. This suggests the existence of relative high-radon events with less than 5% occurrence throughout the year, either in summer or winter, which reach similar maximum hourly concentrations of 74.7 Bq/m^3^ and 71.6 Bq/m^3^ respectively. These high concentrations observed in Winter can be associated with the small vertical development of the nocturnal atmospheric boundary layer.

### 3.2. Baseline and Maximum Daily ^222^Rn Concentrations: Daily Differences

One way to investigate and identify the impact of local and regional contributions to ^222^Rn activity concentrations is to check the variability of radon daily cycles and to analyse the meteorological conditions associated with different amplitudes. The amplitude is defined as the difference between the maximum and minimum daily ^222^Rn concentrations. Therefore, to perform this analysis, the magnitude of the “background” and maximum daily values are needed to be set. The background value represents the starting point upon which the local sources and sinks act to produce the final observed concentration. Due to the location of Bilbao, in a coastal area but distant 16 km to the Cantabrian Sea, ^222^Rn activity concentrations associated with the maritime wind sector are relatively high, and hence, the methodology based on selected hourly atmospheric radon concentration measurements in the maritime wind sector [27] is not applicable in this case. For this reason, and in the present study, we have used the methodology used in [5,24] which is based on taking as reference the average of the hourly ^222^Rn measurements in the afternoon minimum period (16:00–20:00 UTC) (Figure 2) as our daily background reference value at each of the sites.

**Figure 4:**
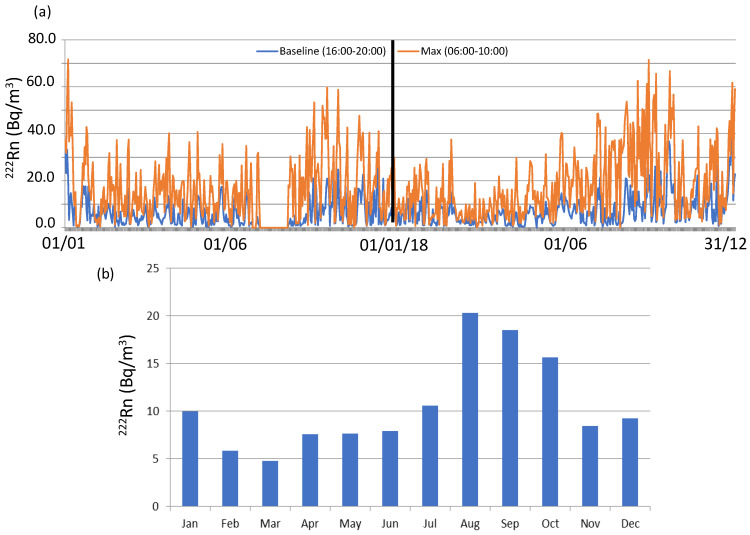
(**a**) Evolution of the ^222^Rn baseline and maximum daily values at Bilbao, considering the average of the afternoon minimum (16:00–20:00 UTC) and morning maximum (06:00–10:00) measurements during the whole sampling period; (**b**) monthly difference between ^222^Rn baseline and maximum daily values.

Figure 4 (orange line) displays the daily ^222^Rn background evolution in Bilbao during the whole sampling period, which has an average of 6.7 ± 0.2 Bq/m^3^. On average, the background value presents low and similar values from February to August (at about 5 Bq/m^3^), while it progressively increases in September-October (9–10 Bq/m^3^) and it reaches the maximum on December 13 Bq/m^3^. This evolution, for instance, is not similar to the one obtained in [5] in Huelva (southern Iberian Peninsula), in which ^222^Rn background increases from May to September, with a decrease from October to November. This difference can be justified according to differences in the sampling sites and the set of meteorological conditions at different scales influencing ^222^Rn activity concentrations. Figure 4 also shows the temporal evolution of the daily maximum ^222^Rn reference values, which have been obtained by averaging the hourly ^222^Rn concentrations in the morning period (06:00–10:00 UTC) (Figure 2). On average, this maximum value progressively increases from March (9.0 Bq/m^3^) to September (27.0 Bq/m^3^), with an average of 17.6 ± 0.6 Bq/m^3^. Similar and high values are registered between August and October, while it decreases in November and rises again in December and January (above 20 Bq/m^3^).

Once defined and calculated, daily differences between background and maximum daily values are obtained. On average during the whole sampling period, this difference is 11.3 Bq/m^3^ and it presents a monthly variability over the whole sampling period. Figure 4 also displays the monthly average of this difference. It is clear the rising tendency from March to August, and how during the warm period the highest values are reached. This evolution can be justified by the progressive increase from May to September in the frequency of mesoscale circulations favoured by the dominance of synoptic stable conditions causing low gradient pressure forcing in this region [21], while, on the other hand, the decrease is likely due to the more unstable meteorological conditions registered from October onwards, with more influence of synoptic conditions.

The variable under study is the diurnal cycle of hourly ^222^Rn concentrations associated with different daily backgrounds and maximum differences. We have grouped each full day of data depending on this difference. ^222^Rn daily cycles were classified into six different ranges using the values of the percentiles of daily differences, i.e., P5 (the lowest values), P25, P50 (the intermediate values), P75, and P95 (the highest values). The ranges are: less than −0.7 Bq/m^3^, from −0.7 to 2.9 Bq/m^3^, from 2.9 to 8.5 Bq/m^3^, from 8.5 to 17.4 Bq/m^3^, from 17.4 to 31.9 Bq/m^3^ and greater than 31.9 Bq/m^3^. The use of percentiles as a reference has been previously used in the characterization of the local, mesoscale and synoptic conditions associated with different ranges of substances in the atmosphere [37,38].

**Figure 5:**
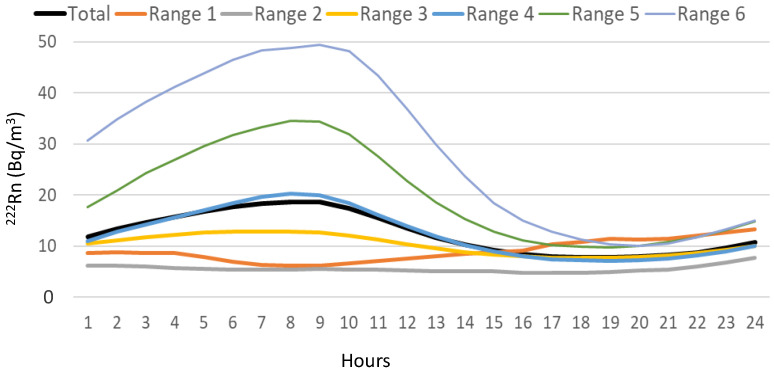
Diurnal composite of average hourly radon concentrations for each range of different daily concentrations in Bilbao.

Figure 5 displays the daily evolution of ^222^Rn concentrations according to each range. The maximum ^222^Rn concentrations increase from 6.1 Bq/m^3^ (Range 2) to 49.5 Bq/m^3^ (Range 6), while the minimum ranges from 4.7 Bq/m^3^ (Range 2) to 11.4 Bq/m^3^ (Range 1). It is interesting to point out how in Range 1 the ^222^Rn concentrations during the daytime are higher than at night, while in the rest of the daily cycles, the concentration of ^222^Rn increases during the night and decreases during the daytime.

### 3.3. Meteorological Link with Daily Cycles: From Local to Synoptic

**Figure 6:**
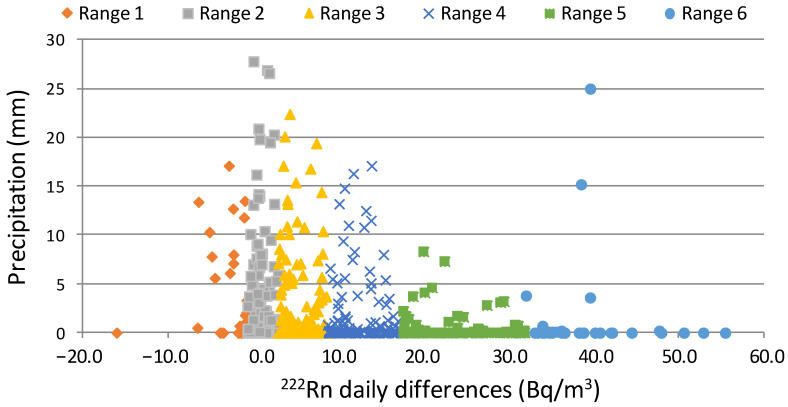
Scatter plot precipitation vs daily differences between maximum and background hourly ^222^Rn concentrations.

The temporal variability of local meteorological factors influences radon activity concentration in the air [39]. Correlation coefficients for daily differences and meteorological parameters were then computed as a measure of the strength of the relationship between daily differences and local meteorological factors [40]. This analysis is based on the set of non-zero pairs between daily radon differences and daily meteorological values, so pairs in which either radon or meteorological values were empty are not included. A total of 645 days (88% of days within the period 2017–2018) were used. A positive correlation with temperature (r = 0.2, at 0.05 significance level (*p* = 4.2 × 10^−7^)) is obtained, which is mainly associated with the occurrence of high temperatures (hence higher radon flux from the soil) in those months with the maximum differences. On the other hand, negative correlations of radon with wind speed (r = −0.3, at 0.05 significance level (*p* = 2.8 × 10^−8^)) and rainfall (r = −0.2, at 0.05 significance level (*p* = 5.6 × 10^−18^)) agree with the dispersive nature of the high wind speed and the decrease in the exhalation due to rainfall. In detail, Figure 6 shows the influence of rainfall episodes with precipitation above 0.1 mm on daily differences of ^222^Rn concentrations. This figure reports that similar daily values are observed under intense rainfall events, which is expected due to rainfall patterns affecting water-table depth and, consequently, soil-moisture content and therefore radon emanations [41]. This figure displays the progressive increase in daily differences between the number of rainy days and the accumulated rainfall.

**Figure 7:**
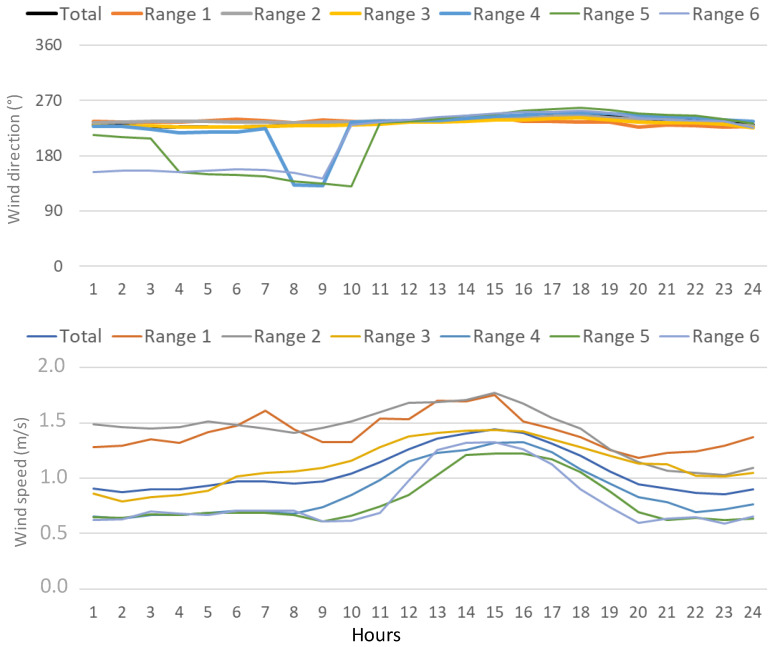
Daily cycles of wind direction (**top**) and speed for each range (**bottom**).

We now investigate the wind dynamics at different scales associated with each type of daily cycle. Figure 7 shows the corresponding daily average wind evolution for each range during the sampling period. This figure displays an evolution from a constant arrival of north-westerly flows associated with Ranges 1–3 to a progressive combination of south-western winds during the morning and north-western flows overnight from range 4 to range 6, i.e., there is a change in surface winds following the increase in ^222^Rn activity concentrations. In this area, one pattern of sea-land breeze has been identified [42]. Briefly, the breeze presents nocturnal flows from the southwest from midnight until midday, followed by the arrival of flows from the northwest overnight. Wind intensity is also following this change in direction, showing a well-marked daily cycle in range 6, while on the contrary, it presents similar daily values in Range 1. On average, wind intensity decreases from 1.4 m/s (Range 1) to 0.8 m/s (Range 6). These conditions would represent a change from near stagnation in the case of Range 1, to a more synoptic influence in the case of Range 6 in this area. In other words, when the wind speed is low, local contributions may be of importance to understanding ^222^Rn activity concentrations, while under strong winds regional transport and distant sources should be considered. This influence of sea-land breezes agrees with previous studies in which this mesoscale circulation plays an important role in the transport and distribution of pollutants [43,44].

**Figure 8:**
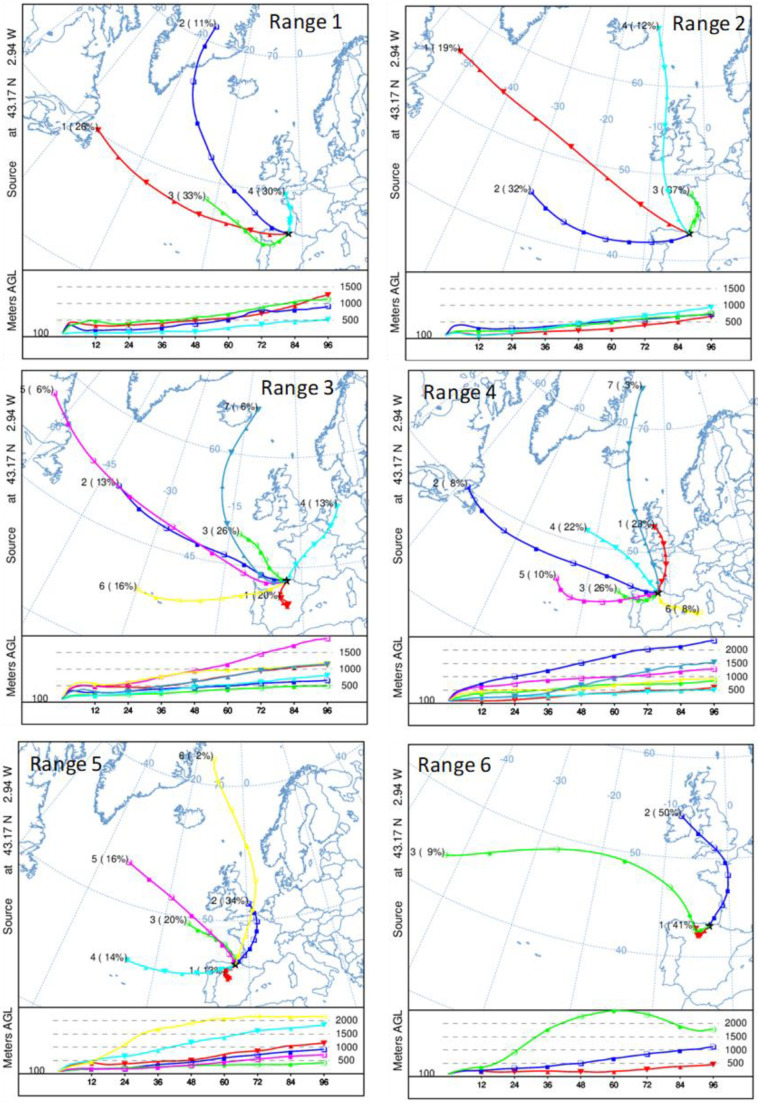
Air mass backward trajectory clusters (computed with HYSPLIT) and their frequencies (in brackets) for each ^222^Rn range.

To complete the analysis, and to identify the synoptic scenarios associated with each daily range, Figure 8 displays the results of the cluster analysis for the 96-h backward trajectory of air mass over Bilbao for each range of ^222^Rn daily differences previously defined. This figure shows the pathway in the horizontal and vertical directions followed by an air parcel upwind from Bilbao. Considering the results in the horizontal direction, in general, the results follow the fact that the higher the daily difference is, the shorter the trajectories are, and the larger displacements over land are present. The air mass analysis for Range 1 indicates the prevalence of westerly flows (70%) with origins and pathways over the Atlantic Ocean, and the arrival of northern circulations, with smaller displacement (30%). The influence of both circulations (westerly and northerly) remains in the backward trajectory cluster associated with Range 2, while in Range 3, air mass clusters representing the arrival of southern air masses and hence with more displacement over land start to be identified. In this range, the arrival of western circulations decreases. Ranges 4–6 point out the continuity in the arrival of westerly and northerly circulations but with less displacement than in previous ranges, i.e., air mass trajectories present less wind velocity. This trend is observed from Range 4 to 6, in which the most influence of nearby circulations (41%) in combination with the arrival of northerly flows (50%) is shown. This combination of airflows, showing the arrival of continental air masses from the northeast and those with small displacement, which agree with the development of mesoscale circulations in this area, and hence, in line with the sea-land breeze pattern identified in Figure 7, are those more influencing in reaching the highest differences in the daily cycles. Therefore, the meteorological scenario mostly influencing reaching the largest daily differences is associated is mostly associated with dry periods, relatively high temperatures and low wind speeds, and two different wind dynamics (1) the arrival of nearby and southerly air masses with the development of sea-land breezes, and (2) arrival of northeast airflow patterns.

### 3.4. Identification of Potential Sources

**Figure 9:**
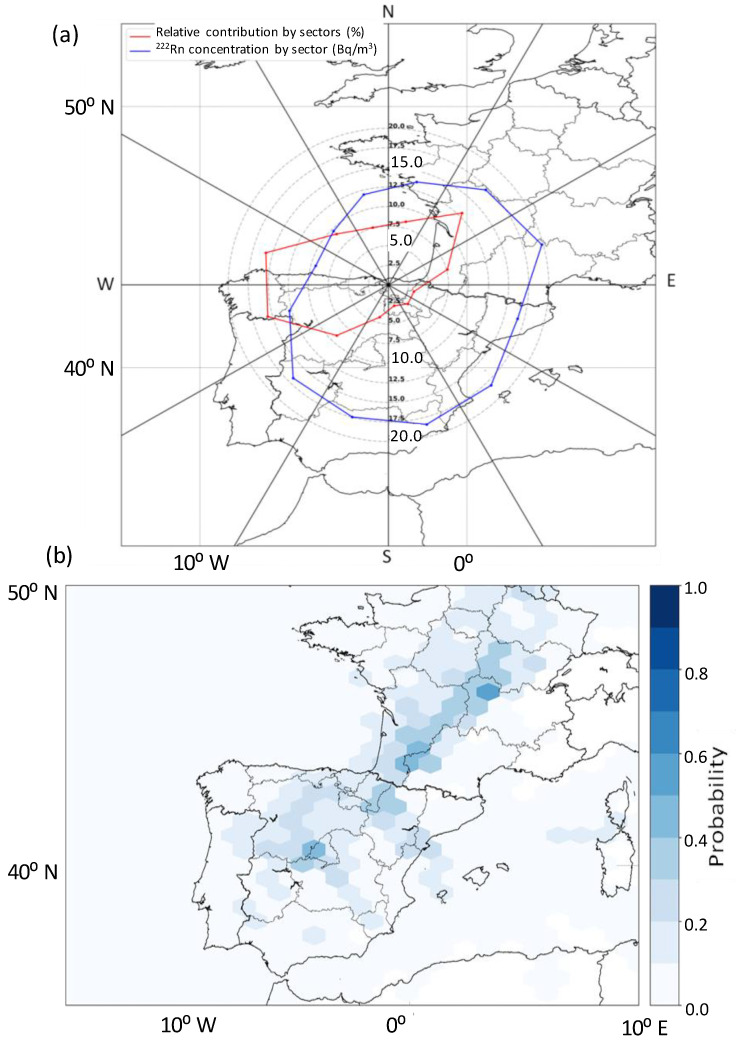
(**a**) Angular concentration and contribution profile for ^222^Rn based on the TSA method, and (**b**) PSCF method identifying those areas more influencing the occurrence of the highest 222Rn activity concentrations at Bilbao.

Once the meteorological scenarios associated with high radon peaks have been defined, this section presents the results obtained in the identification of potential sources of radon over Bilbao. In this sense, an important feature of Bilbao is its proximity to the coast, i.e., coastal sites present lower radon average concentrations than inland sites [14], and the low natural radioactivity levels in this region (Section 2.1). The TSA method is applied by using back trajectories and 3-h ^222^Rn concentrations. This TSA analysis (Figure 9) firstly confirms the main influence of continental winds on the radon concentrations in Bilbao, and secondly, that air masses from the northeast (sectors 2–3) and from the southwest (sectors 8, 9, 10) are mainly associated with the highest radon concentrations, due to their large relative contribution to ^222^Rn measured in Bilbao. These TSA results, then, reflect the highly polluted nature of the combination of southern and north-eastern air mass trajectories over Bilbao, remarking the results of the backward trajectory analysis (Figure 8). There is also a not negligible contribution of pure maritime sectors which could be associated with nearby circulations identified in Figure 8, and hence, associated with mesoscale circulations of sea-land breezes developed in the area.

**Figure 10:**
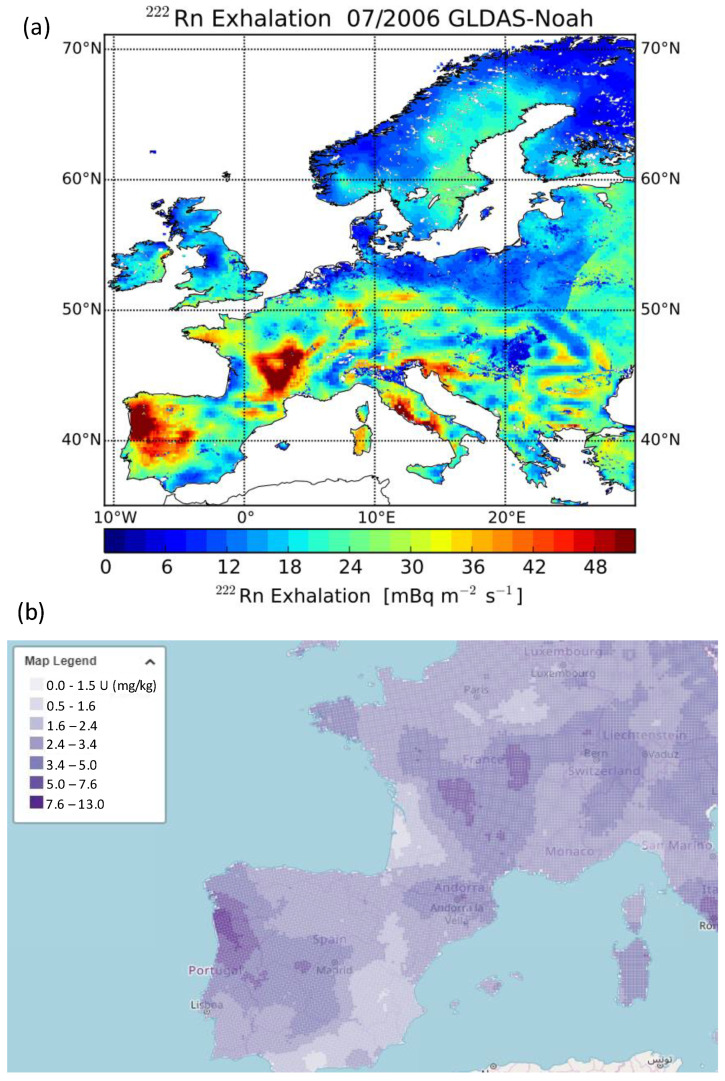
(**a**) ^222^Rn exhalation rate map of European soils, for July 2006 calculated with the monthly mean soil moisture estimates from the GLDAS Noah LSM for July 2006, from [20]; (**b**) Screenshot of the European Map of Uranium in soil available on REMONwebportal (https://remon.jrc.ec.europa.eu/About/Atlas-of-Natural-Radiation/Digital-Atlas/Uranium-in-soil/Uranium-concentration-in-soil-, accessed on 10 October 2022).

Those areas more influencing the highest ^222^Rn concentrations registered at Bilbao, i.e., potential emission sources, are estimated by means of the PSCF model (Figure 9). In the present study, this map reports the areas with the longest residence time of air masses associated with the highest ^222^Rn activity concentrations. Considering previous studies [45,46] in which maximum ABL heights between 950 m in summer and 700 m (13 LT) in winter, and minimum heights of about 100 m were reported, we have only considered for the present analysis those trajectory endpoints at a height lower than 900 m. Figure 9 shows how the potential sources of ^222^Rn in Bilbao are mainly placed in the south, as well as on the French east coast. These areas correspond with the areas with the highest radon flux as well as the area with the highest uranium contents in soil, as it is shown in Figure 10, in which the Radon Flux Map, considering the monthly average for July 2006, and the European Map of Uranium in soil are presented.

Therefore, combining the TSA and PSCF results would present two scenarios in which the highest radon concentrations would be measured in Bilbao. The first one is in which air masses from the northeast could transport radon concentrations from sources placed in the west of France to Bilbao, and the second one is in which south air masses from the south sweep sources placed in the Iberian Peninsula. In both scenarios, the development of sea-land breezes helps in reaching these peak concentrations due to the combination of oceanic air masses which bring markedly smaller concentrations, and continental air masses, which leads to the return of higher ^222^Rn concentrations.

## 4. Conclusions

In this study, local meteorological parameters and air mass backward trajectory cluster analysis, TSA, and PSCF methods were used to investigate local and synoptic meteorological influences on the temporal variability, transport pathways, and potential sources of ^222^Rn in the north of the Iberian Peninsula during the period 2017–2018. ^222^Rn levels were characterized by a diurnal cycle with an early morning maximum and a minimum in the afternoon, although the combination of local and regional meteorological patterns causes differences in seasonal daily patterns, as well as in daily cycle amplitudes. For different daily amplitudes, backward trajectories were analysed. The largest daily amplitude of daily ^222^Rn concentrations is found under the development of mesoscale circulations in this area, which favour the progressive accumulation of ^222^Rn due to the scarce air mass circulation, which agrees with the influence of south and northeast sources. These results are taken as a reference to analyse the great significance of mesoscale circulations in explaining the variability of ^222^Rn, although further investigation is needed to confirm the influence of regional sources on ^222^Rn concentrations and to determine the long-term influence of individual meteorological parameters on radon levels in the air.

## References

[1] Cinelli G., De Cort M., Tollefsen T., European Commission, Joint Research Centre (2019). European Atlas of Natural Radiation.

[2] United Nations Scientific Committee on the Effects of Atomic Radiation (2008). Source and Effects of Ionizing Radiation, Volume I, Annex B.

[3] World Health Organization (2009). Handbook on Indoor Radon.

[4] United Nations Scientific Committee on the Effects of Atomic Radiation (2008). 2006 Report to the General Assembly, with Scientific Annexes. Effects of Ionizing Radiation, Vol. I, Scientific Annex A, Epidemiological Studies of Radiation and Cancer.

[5] Hernández-Ceballos M.A., Vargas A., Arnold D., Bolívar J.P. (2015). The role of mesoscale meteorology in modulating the ^222^Rn concentrations in Huelva (Spain)—Impact of phosphogypsum piles. J. Environ. Radioact..

[6] Vargas A., Arnold D., Adame J.A., Grossi C., Hernández Ceballos M.A., Bolívar J.P. (2015). Analysis of the vertical radon structure at the Spanish “El arenosillo” tower station. J. Environ. Radioact..

[7] Chambers S.D., Williams A.G., Crawford J., Griffiths A.D. (2015). On the use of radon for quantifying the effects of atmospheric stability on urban emissions. Atmos. Chem. Phys..

[8] Levin I., Karstens U., Hammer S., Dellacoletta J., Maier F., Gachkivskyi M. (2021). Limitations of the radon tracer method (RTM) to estimate regional greenhouse gas (GHG) emissions—A case study for methane in Heidelberg. Atmos. Chem. Phys..

[9] Grossi C., Vogel F.R., Curcoll R., Àgueda A., Vargas A., Rodó X., Morguí J.A. (2018). Study of the daily and seasonal atmospheric CH4 mixing ratio variability in a rural Spanish region using ^222^Rn tracer. Atmos. Chem. Phys..

[10] Celikovic I.T., Pantelic G., Vulkanac I., Nikolic J., Zivanovic M., Cinelli G., Gruber V., Baumann S., Quindos L., Rabago D. (2022). Outdoor radon as a tool to estimate radón priority areas—A literature overview. Int. J. Environ. Res. Public Health.

[11] Gutiérrez-Álvarez I., Guerrero J.L., Martón J.E., Adame J.A., Vargas A., Bolívar J.P. (2021). Radon transport events associated with the impact of a NORM repository in the SW of Europe. Environ. Pollut..

[12] Röttger S., Röttger A., Grossi C., Vargas A., Karstens U., Cinelli G., Chung E., Kikaj D., Rennick C., Mertes F. (2022). Radonmetrology for use in climate change observation and radiation protection at the environmental level. Adv. Geosci..

[13] Victor N.J., Singh D., Singh R.P., Singh R., Kamra A.K. (2019). Diurnal and seasonal variations of radon (^222^Rn) and their dependence on soil moisture and vertical stability of the lower atmosphere at Pune, India. J. Atmos. Sol. Terr. Phys..

[14] Arnold D., Vargas A., Vermeulen A.T., Verheggen B., Seibert P. (2010). Analysis of radon origin by backward atmospheric transport modelling. Atmos. Environ..

[15] Hafez Y.I. (2022). Understanding radon (^222^Rn) transport in one and multi-layer soils using analytical and finite element modeling. J. Hydrol..

[16] Yan R., Woith H., Wang R., Wang G. (2017). Decadal radon cycles in a hot spring. Sci. Rep..

[17] Chu B., Liu Y., Ma Q., Ma J., He H., Wang G., Cheng S., Wang X. (2016). Distinct potential aerosol masses under different scenarios of transport at a suburban site of Beijing. J. Environ. Sci..

[18] Li H., He Q., Liu X. (2020). Identification of Long-Range Transport Pathways and Potential Source Regions of PM2.5 and PM10 at Akedala Station, Central Asia. Atmosphere.

[19] Dimitrou K., Kassomenos P. (2020). Background concentrations of benzene, potential long range transport influences and corresponding cancer risk in four cities of central Europe, in relation to air mass origination. J. Environ. Manag..

[20] Karstens U., Schwingshackl C., Schmithüsen D., Levin I. (2015). A process based ^222^Radon flux map for Europe and its comparison to long-term observations. Atmos. Chem. Phys..

[21] Alegría N., Hernández-Ceballos M.A., Herranz M., Idoeta R., Legarda F. (2020). Meteorological Factors Controlling ^7^Be Activity Concentrations in the Atmospheric Surface Layer in Northern Spain. Atmosphere.

[22] Alegría N. (2008). Tesis: Desarrollo de Niveles de Alarma y Análisis de Transitorios en Estaciones de Vigilancia Radiológica Automática. https://dialnet.unirioja.es/servlet/tesis?codigo=212064.

[23] Su L., Yuan Z., Fung Y.C.H., Lau A.K.H. (2015). A comparison of HYSPLIT backward trajectories generated from two GDAS datasets. Sci. Total Environ..

[24] Chambers S.D., Williams A.G., Zahorowski W., Griffiths A., Crawford J. (2011). Separating remote fetch and local mixing influences on vertical radon measurements in the lower atmosphere. Tellus B.

[25] Zimnoch M., Wach P., Chmura P., Gorczyca Z., Rozanski K., Godlowska J., Mazur J., Kozak K., Jericevic A. (2014). Factors controlling temporal variability of near-ground atmospheric ^222^Rn concentration over central Europe. Atmos. Chem. Phys..

[26] Chambers S.D., Galeriu D., Williams A.G., Melintescu A., Griffiths A.D., Crawford J., Dyer L., Duma M., Zorila B. (2016). Atmospheric stability effects on potential radiological releases at a nuclear research facility in Romania: Characterising the atmospheric mixing state. J. Environ. Radioact..

[27] Zahorowski W., Chambers S.D., Wang T., Kang C.H., Uno I., Poon S., Oh S.N., Wcrczynski S., Kim J., Henderson-Sellers A. (2005). Radon-222 in boundary layer and free tropospheric continental outflow events at three ACE-ASIA sites. Tellus B.

[28] Jorba O., Pérez C., Rocadenbosch F., Baldasano J.M. (2004). Cluster analysis of 4-day back trajectories arriving in the Barcelona area, Spain, from 1997 to 2002. J. Appl. Meteorol..

[29] Baeza A., Corbacho J.A., Rodríguez A., Galván J., García-Tenorio R., Manjón G., Mantero J., Vioque I., Arnold D., Grossi C. (2012). Influence of the Fukushima Dai-ichi nuclear accidento on Spanish environmental radioactivity levels. J. Environ. Radioact..

[30] Hernández-Ceballos M.A., Adame J.A., Bolívar J.P., De la Morena B.A. (2013). Vertical behavior and meteorological properties of air masses in the southwest of the Iberian Peninsula (1997–2007). Meteorol. Atmos. Phys..

[31] Izquierdo R., Alarcón M., Aguillaume L., Àvila A. (2014). Effects of teleconnection patterns on the atmospheric routes, precipitation and deposition amounts in the north-eastern Iberian Peninsula. Atmos. Environ..

[32] Lee S., Ashbaugh L. (2007). The impact of trajectory starting heights on the MURA trajectory source apportionment (TSA) method. Atmos. Environ..

[33] Li M., Huang X., Zhu L., Li J., Song Y., Cai X., Xie S. (2012). Analysis of the transport pathways and potential sources of PM10 in Shanghai based on three methods. Sci. Total Environ..

[34] Fleming Z.L., Monks P.S., Manning A.J. (2012). Review: Untangling the influence of air-mass history in interpreting observed atmospheric composition. Atmos. Res..

[35] Hondula D.M., Davis R.E. (2011). Decline in wintertime air-mass transition frequencies in the USA. Clim. Res..

[36] Vinuesa J.F., Basu S., Galmarini S. (2007). The diurnal evolution of ^222^Rn and its progeny in the atmospheric boundary layer during the Wangara experiment. Atmos. Chem. Phys..

[37] Lozano R.L., Hernández-Ceballos M.A., San Miguel E.G., Adame J.A., Bolívar J.P. (2012). Meteorological factors influencing on surface air 7Be and 210Pb concentrations from southwestern Iberian Peninsula. Atmos. Environ..

[38] Brattich E., Hernández-Ceballos M.A., Cinelli G., Tositti L. (2015). Analysis of 210Pb peak values at Mt. Cimone (1998–2011). Atmo. Environ..

[39] Fujiyoshi Y., Yamashita K., Fujiwara C. (2006). Visualization of streaks, thermals and waves in the atmospheric boundary layer. J. Vis..

[40] Baciu A.C. (2005). Radon and thoron progeny concentration variability in relation to meteorological conditions at Bucharest (Romania). J. Environ. Radioact..

[41] Griffiths A.D., Zahorowski W., Element A., Werczynski S. (2010). A map of radon flux at the Australian land surface. Atmos. Chem. Phys..

[42] Hernández-Ceballos M.A., Legarda F., Alegría N. (2020). Analysis of Alpha Activity Levels and Dependence on Meteorologycal Factors over a Complex Terrain in Northern Iberian Peninsula (2014–2018). Int. J. Environ. Res. Public Health.

[43] Miao T., Pan T. (2015). A multiphysics model for evaluating electrokinetic remediation of nuclear waste-contaminated soils. Water Air Soil Pollut..

[44] Zhang X., Ma W., Wu L. (2019). Effect of Mesoscale Oceanic Eddies on Extratropical Cyclogenesis: A tracking Approach. JRG Atmos..

[45] González-Aparicio I., Hidalgo J. (2012). Dynamically based future daily and seasonal temperatura scenarios analysis for the northern Iberian Peninsula. Int. J. Climatol..

[46] Arrillaga J.A., Yagüe C., Sastre M., Román-Cascón C. (2016). A characterisation of sea breeze events in the Eastern Cantabrian coast (Spain) from observational data and WRF simulations. Atmos. Res..

